# Supplementary material for: Chromothripsis during telomere crisis is independent of NHEJ, and consistent with a replicative origin
Source: Genome Res. 2019 May;29(5):737–49. doi: 10.1101/gr.240705.118 (PMC6499312; doi:10.1101/gr.240705.118)
Supplement: Supplemental Material [file supp_gr.240705.118_Supplemental_file_1.zip › contigs/annotated_contigs/DB99/contig.2.DB99_length_329_mean_cov_5.01519756839.docx]

**DB99_length_329_mean_cov_5.01519756839**

```
ACTAGTATGAAATATTTTAGGGCACAAAAAACTGCACTCATATAAGACAATGGACTTAATCAATAAATATTGTGTGTGTTCTGACTACT
 >chr12:126705456-126705616 + E=5e-85
CTACCAACTGGCCATTTCCCCATCCCTGTCCCTCTTCTAAGACTTTTTATTCCCTGAGACTACACAGTAA|T|TACACACACACACAGA
                                                                       >chr12:126999980
CACACACACGCATACACACGGTTTGGCTCTGAAAGTAGTAATTTTCCTTTCTTCTATATGTCCATTGAAATCATTCAAGACGCTCAAGA
-127000150 + E=5e-88
CACATTTAGCAATGGTGGATGGCAAAATGAAGGATAACAGGGGTTATTTAAGTTAAACATGCAT
```
